# Multi-Modality Imaging for Interventions in Tricuspid Valve Disease

**DOI:** 10.3389/fcvm.2021.638487

**Published:** 2021-02-09

**Authors:** Federico Fortuni, Kensuke Hirasawa, Jeroen J. Bax, Victoria Delgado, Nina Ajmone Marsan

**Affiliations:** Department of Cardiology, Leiden University Medical Center, Leiden, Netherlands

**Keywords:** tricuspid regurgitation, transcatheter tricuspid valve repair, multimodality imaging, echocardiography, cardiac magnetic resonance, multidetector row computed tomography, tricuspid valve

## Abstract

Several studies have demonstrated that severe tricuspid regurgitation (TR) has a significant negative impact on morbidity and mortality. Nowadays, several therapeutic options to treat TR are available and patients at high surgical risk can also be treated with transcatheter procedures. For the management of patients with TR, an accurate assessment of the tricuspid valve and its surrounding structures is therefore of crucial importance and has gained significant interest in the medical community. Different imaging modalities can provide detailed information on the tricuspid valve apparatus, right ventricle, right atrium, and coronary circulation which are fundamental to define the timing and anatomic suitability of surgical and percutaneous procedures. The present review illustrates the role of 2D and 3D echocardiography, cardiac magnetic resonance, and multidetector row computed tomography for the assessment of the tricuspid valve and right heart with a particular focus on the data needed for planning and guiding interventional procedures.

## Introduction

The tricuspid valve (TV) apparatus is a complex structure consisting of three leaflets inserted in the tricuspid annulus (TA) and connected to the papillary muscles of the right ventricle (RV) through the chordae tendinae (1). The function of the TV depends on the integrity of its apparatus and surrounding structures, the RV and the right atrium (RA). The most common form of TV dysfunction is tricuspid regurgitation (TR), which is often a mild, benign condition. However, hemodynamically significant (≥ moderate) TR is also quite frequent with an estimated prevalence of ~1.6 million individuals in the United States (2). The mechanisms underlying TR can be grouped into two categories: primary and secondary (3). Primary TR is caused by alterations directly affecting the valve structures including leaflets, chordae and papillary muscles. Conversely in secondary TR, valve structures are intact but their motion is altered due to RV and/or RA dysfunction. In the past, significant TR was largely neglected and undertreated (4) due to the poor outcomes associated with isolated TV surgery (5) and the false belief that TR did not affect patient prognosis. More recently, several studies have demonstrated that TR can independently worsen patient outcomes and quality of life if left untreated (6–8). Furthermore, the introduction of transcatheter procedures for the TV have opened the possibility to treat also patients at high surgical risk and have increased awareness on TR and the interest on the non-invasive assessment of the TV.

Current American and European guidelines recommend TV interventions (preferably TV repair) at the time of left-sided valve surgery in case of severe TR (class I) or in the presence of TA dilation regardless of TR severity (class IIa) (9, 10). On the contrary, TV intervention is recommended as a stand-alone procedure in the case of symptomatic severe primary TR or asymptomatic severe primary TR with progressive RV dysfunction (class IIb) (9, 10). In the presence of secondary TR, TV intervention should be considered in symptomatic severe TR in the absence of severe left ventricular (LV) or RV dysfunction and of severe pulmonary hypertension (class IIa) (9, 10). Specific TV transcatheter procedures are currently not included in the guidelines but there is growing evidence of their safety and efficacy in high surgical risk patients (4). These interventions however require specific criteria of anatomic feasibility and information to plan the procedure (11–13). Different imaging modalities play therefore a major role in providing all the data needed in these various procedures, namely to define the optimal timing, the most suitable approach and to prevent potential complications during the intervention. In this review, we will illustrate the value of multi-modality imaging in the comprehensive assessment of patients with TR and to effectively plan and monitor TV interventions.

## Anatomy of the Tricuspid Valve and the Right Heart

**Figure 1:**
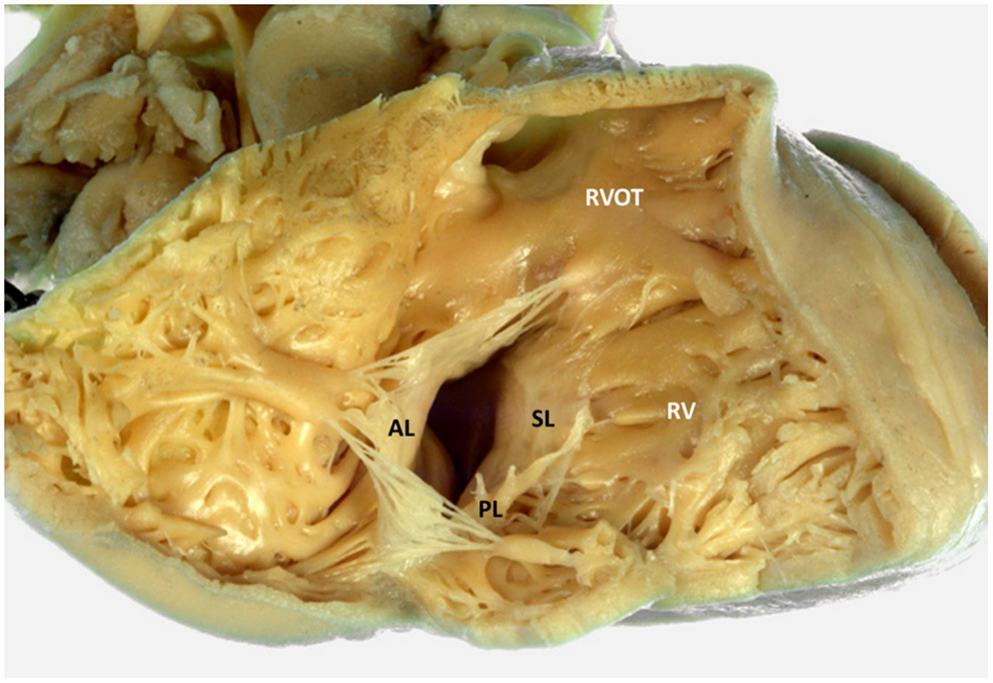
Tricuspid valve and right ventricular anatomy. The figure displays an anatomic specimen with a view of the tricuspid valve from the right ventricle (courtesy of Dr. Monique R.M. Jongbloed and Ing. L.J. Wisse, Department of Cardiology and Department of Anatomy and Embryology, Leiden University Medical Center, Leiden, The Netherlands). AL, anterior leaflet; RV, right ventricle; RVOT, right ventricular outflow tract; PL, posterior leaflet; SL, septal leaflet.

The TV is the atrioventricular valve located between the RA and RV (Figure 1) with a more apical position as compared to the mitral valve. The TV is a complex and dynamic structure that usually consists of three leaflets inserted on the TA. The three TV leaflets are thinner as compared to the two mitral valve leaflets and are named according to their anatomical position (Figure 1): anterior, septal and posterior leaflet (14). The anterior leaflet is the largest and has a quadrangular shape. The posterior leaflet is the smallest and has a triangular shape, while the septal leaflet has a semicircular shape that follows the orientation of the interatrial and interventricular septum (15). The TA has a dynamic saddle-shape (16), characterized by having the higher points on the antero-septal and postero-lateral portions and the lower ones in the antero-lateral and postero-septal portions (17). The TA dimensions vary throughout the cardiac cycle being overall larger during diastole and smaller during systole. The shape of the TA tends to be more oval in patients without significant TR and becomes more circular in patients with severe TR (16). The TA has important spatial relations with several surrounding structures. The antero-septal portion of the TA is in close spatial relationship with the right and non-coronary sinus of the aorta. The right coronary artery (RCA), which usually arises from the right coronary sinus, runs across the atrioventricular groove close to the anterior and posterior aspects of the TA; particularly, in its anterior course the RCA can be very close to the TA (18). In up to 7–8% of individuals, the RCA runs within 2 mm of the anterior TA which makes this location critical for the risk of compression or permanent damage during surgical or transcatheter TV annuloplasty (19). The atrial caval tricuspid isthmus is the area between the inferior vena cava and the TV. This region is highly variable and can present a large Eustachian ridge, the Chiari's network (20) and aneurismal pouches that can affect the maneuverability of the catheters in the RA. The postero-septal portion of the TA is limited by the coronary sinus that together with the TA and the anteriorly located atrioventricular node demarcate the triangle of Koch. The atrioventricular node is located superiorly to the antero-septal portion of the TA and continues caudally with the His bundle that is in a close spatial relation with the antero-septal portion of the TA. Therefore, compressions at this level may cause a complete atrioventricular block and, in case of a permanent injury, the need for permanent pace-maker implantation (21). The TA separates the RA from the RV and therefore is affected by both these structures, as both RA and/or RV dilatation can determine TA enlargement (22). The TV leaflets are connected to the papillary muscles of the RV through multiple chordae tendinae. RV dysfunction and remodeling can therefore affect the correct function of the TV through multiple mechanisms. The dilation of the RV can determine the apical displacement of the papillary muscles that alter the angulation and pull the chordae tendinae and the corresponding TV leaflets during systole, resulting in leaflet tenting and a so-called type IIIb TR (restricted systolic motion) according to the Carpentier classification. Moreover, also presence of dyssynchrony in the contraction of the papillary muscles during systole can determine a malcoaptation of the TV leaflets and cause TR (23, 24).

## Imaging of the Tricuspid Valve and Identification of TR Etiology

**Figure 2:**
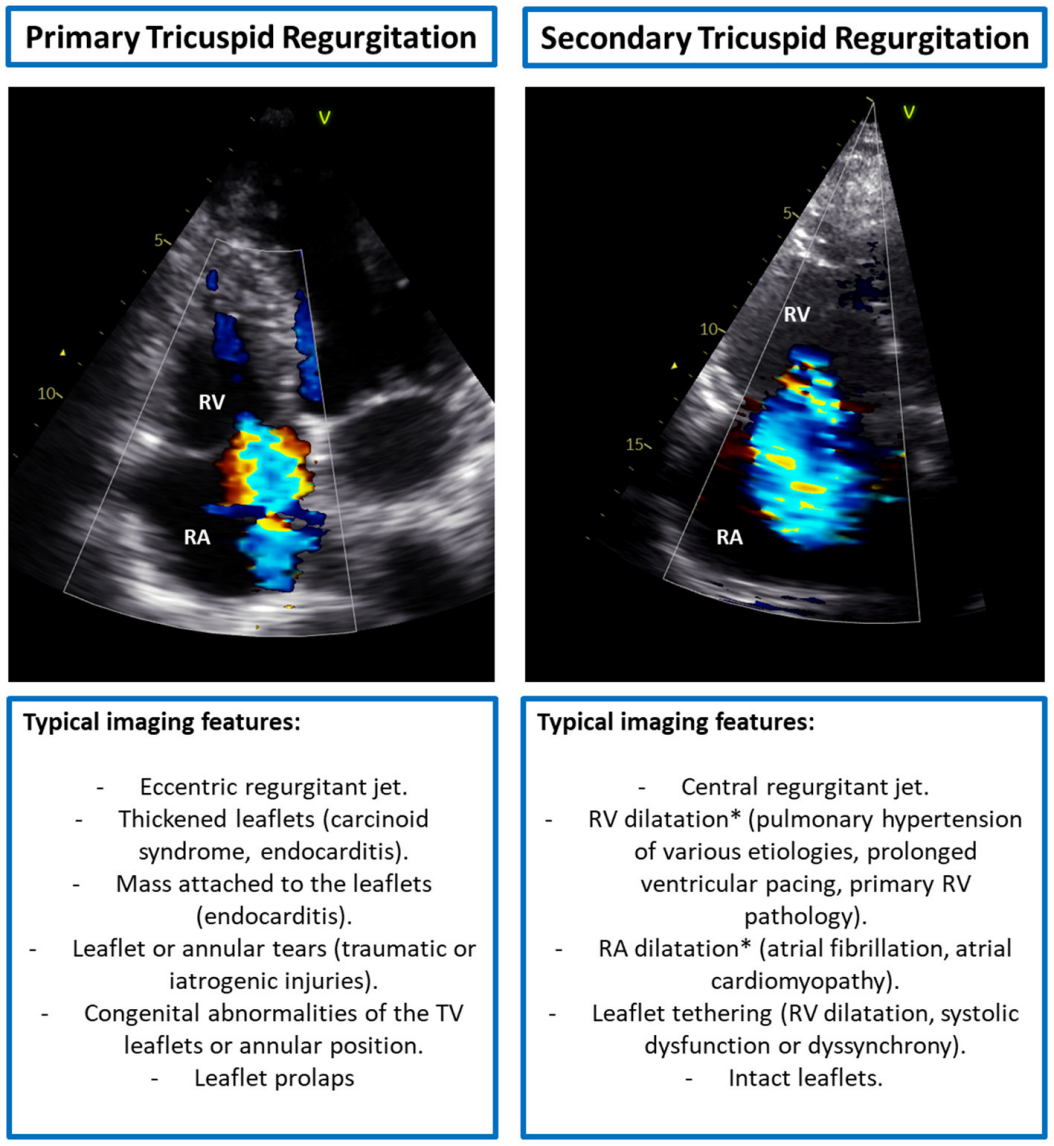
Typical imaging features of primary vs. secondary tricuspid regurgitation. *Both RV and RA dilatation can cause TA dilatation. RA, right atrium; RV, right ventricle; TA, tricuspid annulus; TV, tricuspid valve.

The identification of the potential mechanisms underlying TR represents the first step in the evaluation of patients with TR. The causes of TR can be grouped into two main categories, primary TR and secondary TR (3) (Figure 2). Primary TR accounts for <10% of the cases and depends on organic alterations of the leaflets or sub-valvular apparatus that can be secondary to acquired diseases, congenital abnormalities, traumatic or iatrogenic injuries. The most common causes are (i) endocarditis, which is frequent in intravenous drug users or immunodepressed patients following invasive procedures; (ii) iatrogenic, following invasive procedures on the right heart such as endomyocardial biopsy or pace-maker lead implantation; (iii) leaflet prolapse, that can also be associated with mitral valve prolapse in patients with myxomatous valve disease; (iv) carcinoid syndrome, where the TV leaflets appear significantly thickened due to extracellular matrix deposition (25); and (v) rheumatic heart disease, that is quite rare in developed countries whereas it is among the most frequent causes of primary TR in developing countries. Secondary TR represents the most common type of TR accounting for more than 90% of the cases. In secondary TR, the TV and sub-valvular apparatus are intact but there is a malcoaptation of the leaflets due to TA enlargement and/or RV dilatation or dysfunction that causes tethering of the leaflets.

**Figure 3:**
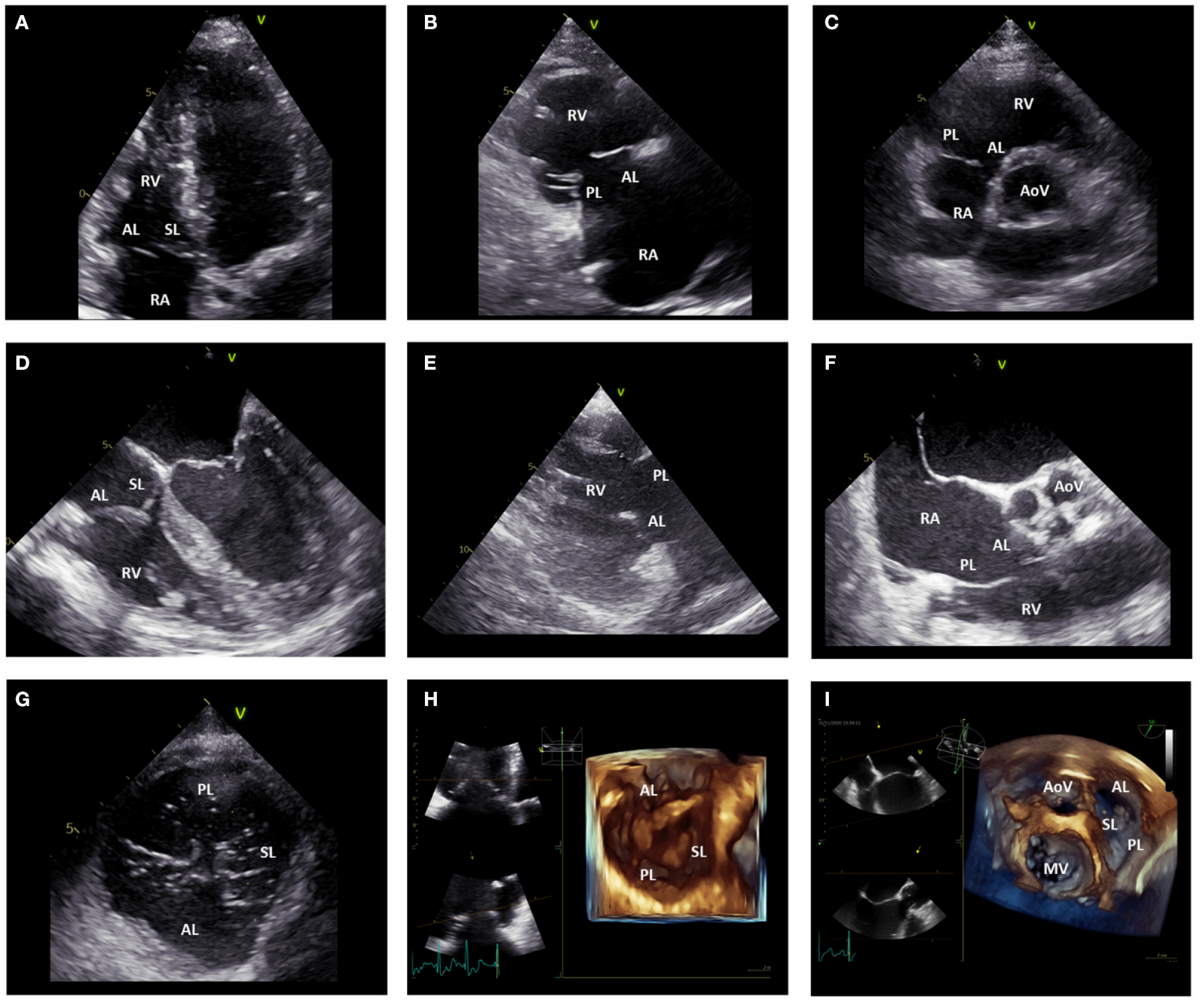
Echocardiographic assessment of the tricuspid valve. **(A)** displays the apical four-chamber view where the anterior and septal leaflet of the TV can be visualized. **(B)** shows the parasternal long-axis view of the RV where the anterior and posterior leaflet of the TV can be imaged. In the parasternal short-axis view **(C)**, the posterior and anterior leaflet of the TV can be imaged. **(D)** shows the mid-esophageal four-chamber view where the anterior and septal leaflet of the TV can be observed. **(E)** displays the transgastric RV inflow view where the posterior and anterior leaflet of the TV can be imaged. From the mid-esophageal RV inflow-outflow view **(F)** the anterior and posterior leaflet of the TV can be imaged. From the transgastric TV short-axis view **(G)**, all the TV leaflets can be simultaneously visualized. Similarly, 3D images of the TV acquired from the apical **(H)** and mid-esophageal **(I)** four-chamber view can show all the TV leaflets simultaneously. AL, anterior leaflet; AoV, aortic valve; MV, mitral valve; PL, posterior leaflet; RA, right atrium; RV, right ventricle; SL, septal leaflet; TV, tricuspid valve.

Transthoracic two-dimensional (2D) echocardiography is the pivotal imaging modality to evaluate the TV apparatus, RA and RV due to its wide availability. Echocardiography provides fundamental information regarding the anatomy and specific lesions of the leaflets and whole valvular apparatus, including surrounding structures which are very important to identify the etiology of TR (26). Considered the irregular crescent shape of the RV and the complex, non-planar structure of the TV, more views are necessary to perform a comprehensive assessment of the TV (26, 27) (Figure 3). The three leaflets can only occasionally be visualized simultaneously with 2D transthoracic echocardiography (either from the short-axis view or subcostal view) and dedicated views should be used to identify (in relation to the surrounding structures) each leaflet. In the parasternal long-axis view of the RV, the anterior leaflet is always visible in the upper part of the sector, whereas in the lower portion of the image either the septal leaflet can be seen if the interventricular septum is in view, or the posterior leaflet if the coronary sinus is in view. In the parasternal short-axis view at the level of the aortic valve, the entire anterior leaflet or the posterior and anterior leaflets can be imaged. In the apical 4-chamber view, the septal leaflet can always be observed close to the septum, whereas the opposing leaflet can be either the anterior leaflet (if the probe is angulated anteriorly through the aortic valve) or the posterior leaflet (if the probe is angulated posteriorly through the coronary sinus). Nevertheless, the TV leaflets have large anatomic variability and bicuspid/quadricuspid TV may be occasionally encountered. In this cases, the identification of the TV leaflets with 2D echocardiography have important limitations. On the contrary, three-dimensional (3D) echocardiography allows for the systematic simultaneous visualization of the TV leaflets and their commissures (antero-septal, antero-posterior, and postero-septal), may be useful to clarify difficult anatomies and can be acquired both by the transthoracic and transesophageal approach, using a dedicated acquisition. The imaging of the TV with transesophageal echocardiography can be challenging due to the anterior location, far from the probe, of the TV. However, transesophageal echocardiography is fundamental for intraprocedural monitoring/guidance and to clarify dubious findings at the transthoracic approach. Several transesophageal views can be used to image the different leaflets (Figure 3). In the mid-esophageal four-chamber view, the anterior and septal leaflets can be visualized. In the mid-esophageal RV inflow-outflow view, the anterior and posterior leaflets can be imaged. In the transgastric short-axis view at the level of the TV, all three leaflets can be simultaneously imaged; whereas the transgastric RV inflow view allows for the simultaneous visualization of the posterior and anterior leaflets. Furthermore, when using 3D acquisition bi-plane views can also be used allowing for simultaneous visualization of the 3 TV leaflets.

**Figure 4:**
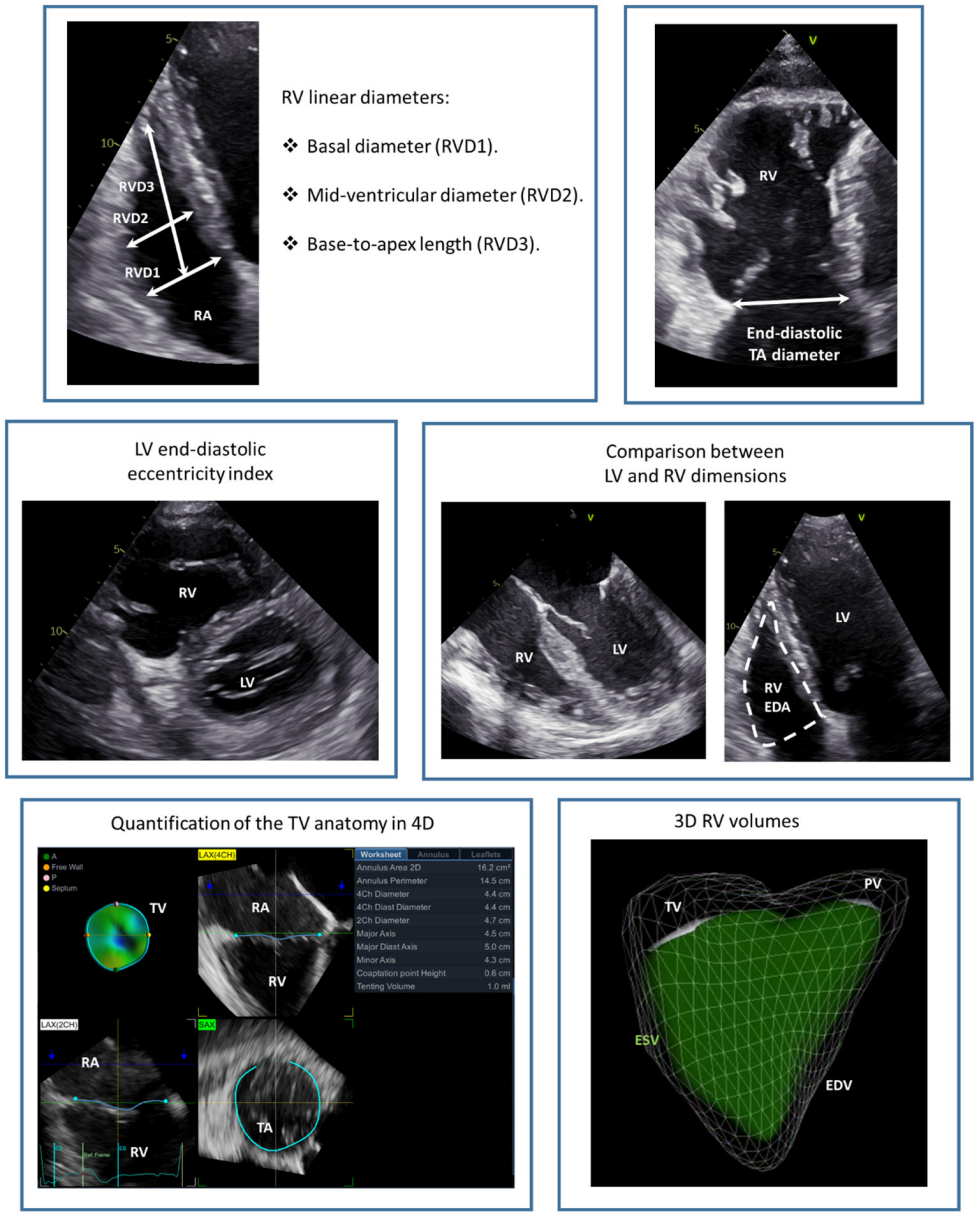
Echocardiographic assessment of the tricuspid annulus and right ventricular dimensions. The figure presents a summary of the main echocardiographic measurements of RV and TA dimensions. EDA, end-diastolic area; EDV, end-diastolic volume; ESV, end-systolic volume; LV, left ventricle; PV, pulmonary valve; RA, right atrium; RV, right ventricle; RVD, right ventricular diameter; TA, tricuspid annulus; TV, tricuspid valve.

The TA diameter is an important parameter and can be measured from the transthoracic apical or trans-esophageal mid-esophageal four-chamber view (Figure 4). This measurement represents the distance between the hinge points of opposing leaflets of the TV at an end-diastolic frame. A TA diameter ≥ 40 mm or 21 mm/m^2^ is indicative of TA dilatation. In secondary TR, the TA dilates more posteriorly and its shape becomes more circular as TR becomes more severe. Accordingly, it had been demonstrated that 2D echocardiography systematically underestimates the TA diameter compared to a 3D assessment (28). The tenting of the TV leaflets can also be quantified with 2D echocardiography by measuring: (i) the tenting area, which is the area between the TV leaflets and the TA; and (ii) the tenting height that represents the distance between the TV leaflets coaptation point and the TA (29). These parameters should be both measured at mid-systole and from an apical 4-chamber view focused on the RV. Moreover, 3D echocardiography allows also for the measurement of TV tenting volume that showed to be related to residual TR after TV annuloplasty (30).

**Figure 5:**
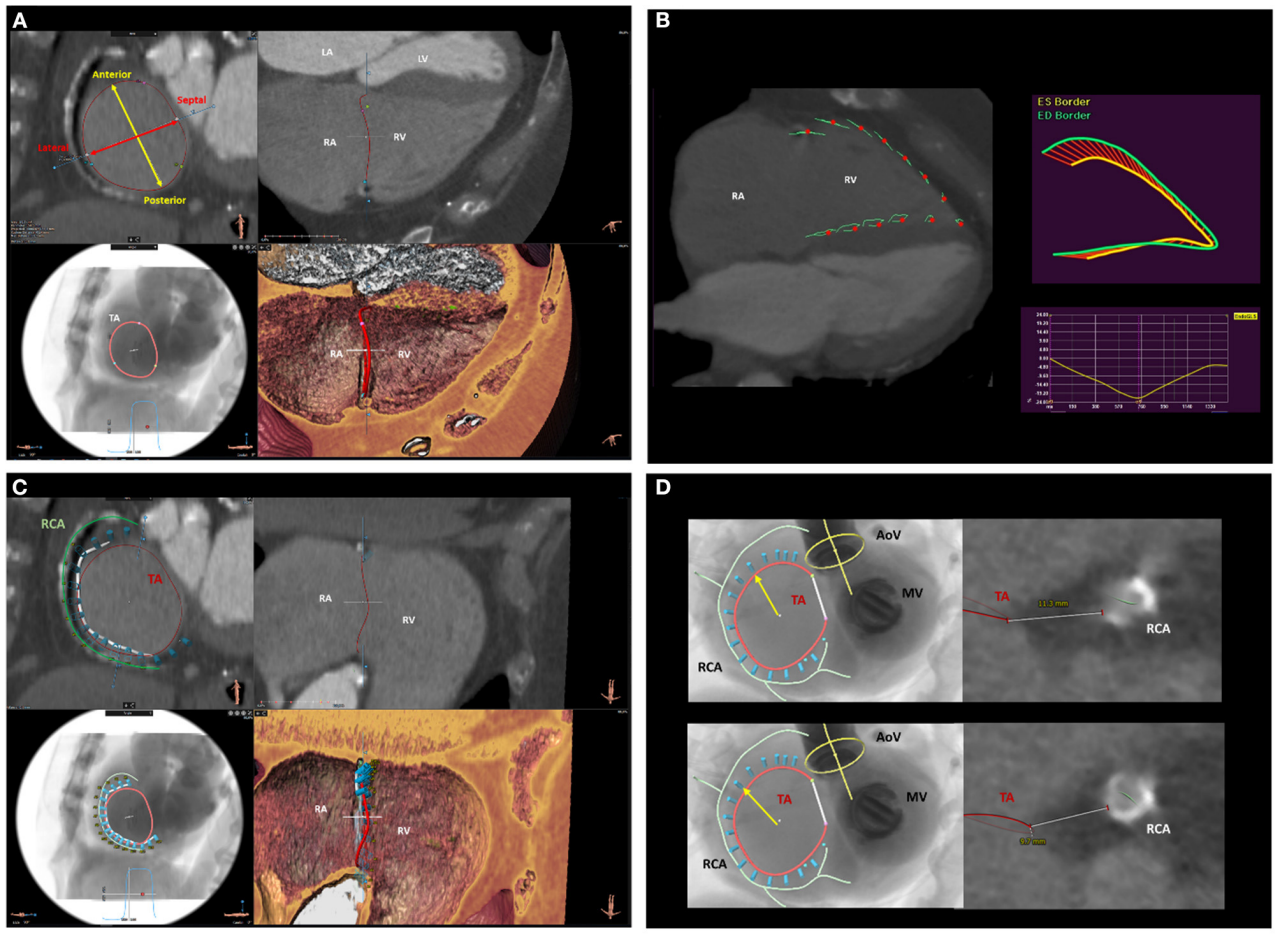
Multi-detector row computed tomography assessment of the tricuspid valve and surrounding structures. **(A)** displays the MDCT assessment of TA anatomy and dimensions. **(B)** shows the functional assessment of the RV using computed tomography feature-tracking longitudinal strain. **(C)** shows the spatial relation of the TA (red circle) with the RCA (green line). This is an important information to assess the feasibility and plan the location where to implant the anchors (light blue cylinders) of the Cardioband device. **(D)** shows the assessment of the distance between the RCA and the TA on two predicted positions where the anchors of the Cardioband device will be placed. LA, left atrium; LV, left ventricle; MDCT, multi-detector row computed tomography; RA, right atrium; RCA, right coronary artery; RV, right ventricle; TA, tricuspid annulus.

Multi-detector row computed tomography (MDCT) is characterized by high spatial resolution which makes this imaging modality ideal to assess the TA dimensions and the relationship of the TA with the RCA which are fundamental to plan interventional procedures on the TA (i.e., transcatheter TV annuloplasty) (Figure 5). The quality of the images is of utmost importance and depends on image acquisition which may be challenging due to the frequent presence of atrial fibrillation and intracardiac devices which may be associated with TR. Moreover, patients with chronic kidney disease are at higher risk of developing contrast-induced acute kidney injury which can be prevented by prophylactic administration of intravenous normal saline and by limiting the use of contrast media. Dedicated protocols exist to study the TV (31), limit the use of contrast media, avoid artifacts and provide a homogeneous opacification of the right heart. A dedicated post-processing software of MDCT data allows also to place a virtual transesophageal probe at the desired level in the esophagus, adjust the ante-/retro-flexion, right/left flexion and rotational degree to reproduce the echocardiographic views that would be needed to visualize a specific structure during the procedure (32, 33).

**Figure 6:**
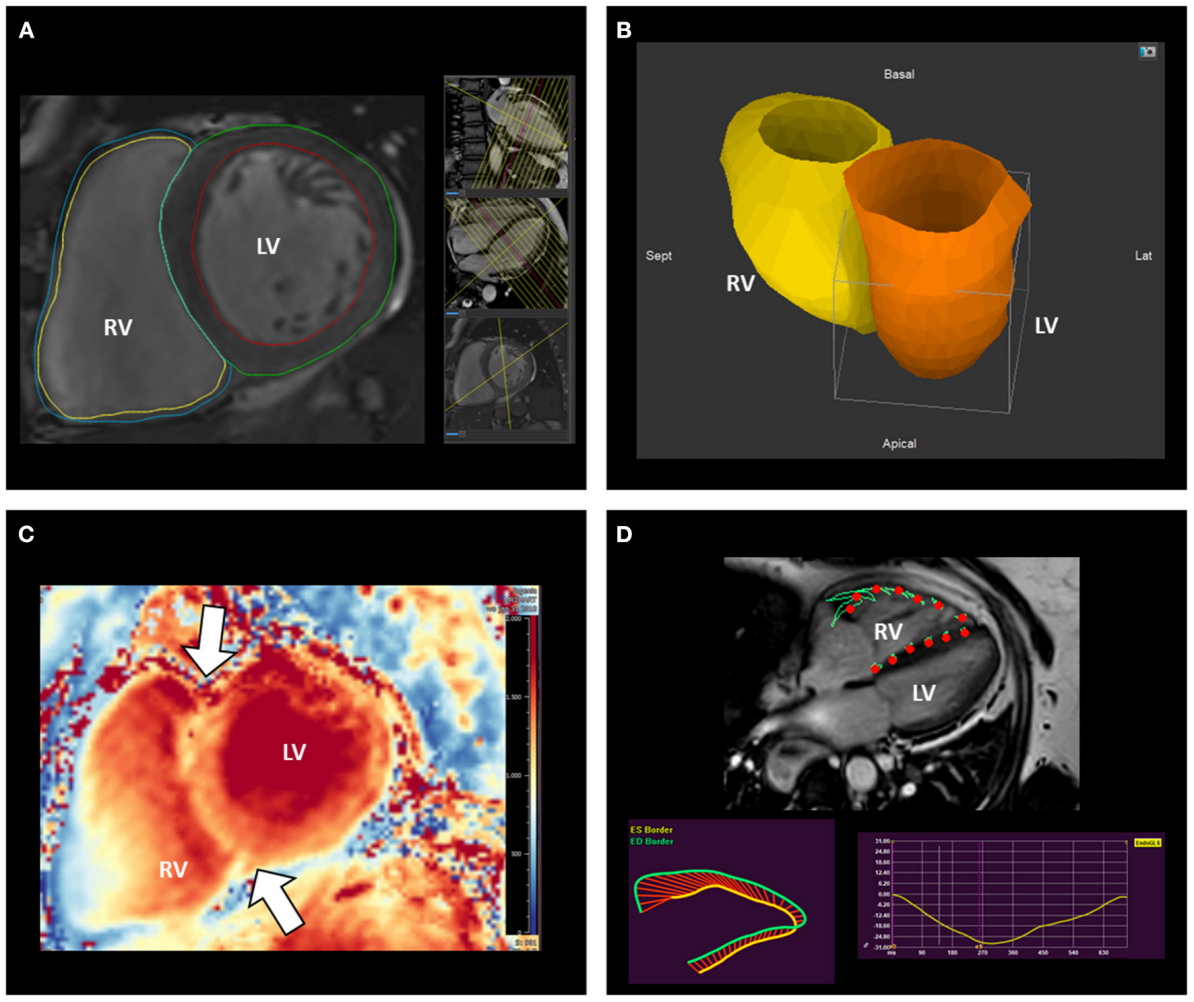
Assessment of right ventricular remodeling with cardiovascular magnetic resonance. CMR represents the gold standard to measure RV volumes **(A,B)** and mass **(A)**. The analysis of T1 mapping allows also the investigation of the stress-induced remodeling by the RV on its anterior and posterior insertion points (arrows) on the interventricular septum **(C)**. **(D)** illustrates the assessment of RV systolic function using CMR feature-tracking RV free-wall and global longitudinal strain. CMR, cardiac magnetic resonance; LV, left ventricle; RV, right ventricle.

Cardiac magnetic resonance (CMR) is the gold standard imaging modality to evaluate RV size and systolic function without the use of contrast media (Figure 6). However, being the TV leaflets very thin, CMR is not very suited for their assessment. The absence of non-compatible MR devices should be checked before performing the exam and intracardiac devices should be programmed in an MR compatible mode. CMR image acquisition in patients with atrial fibrillation may be challenging; nevertheless, accurate measurements can be achieved using real-time cine sequences (34). The presence of RA or RV dilatation, leaflet tethering or malcoaptation can be observed and provide indications on the potential etiology of TR.

## Grading of TR Severity

**Figure 7:**
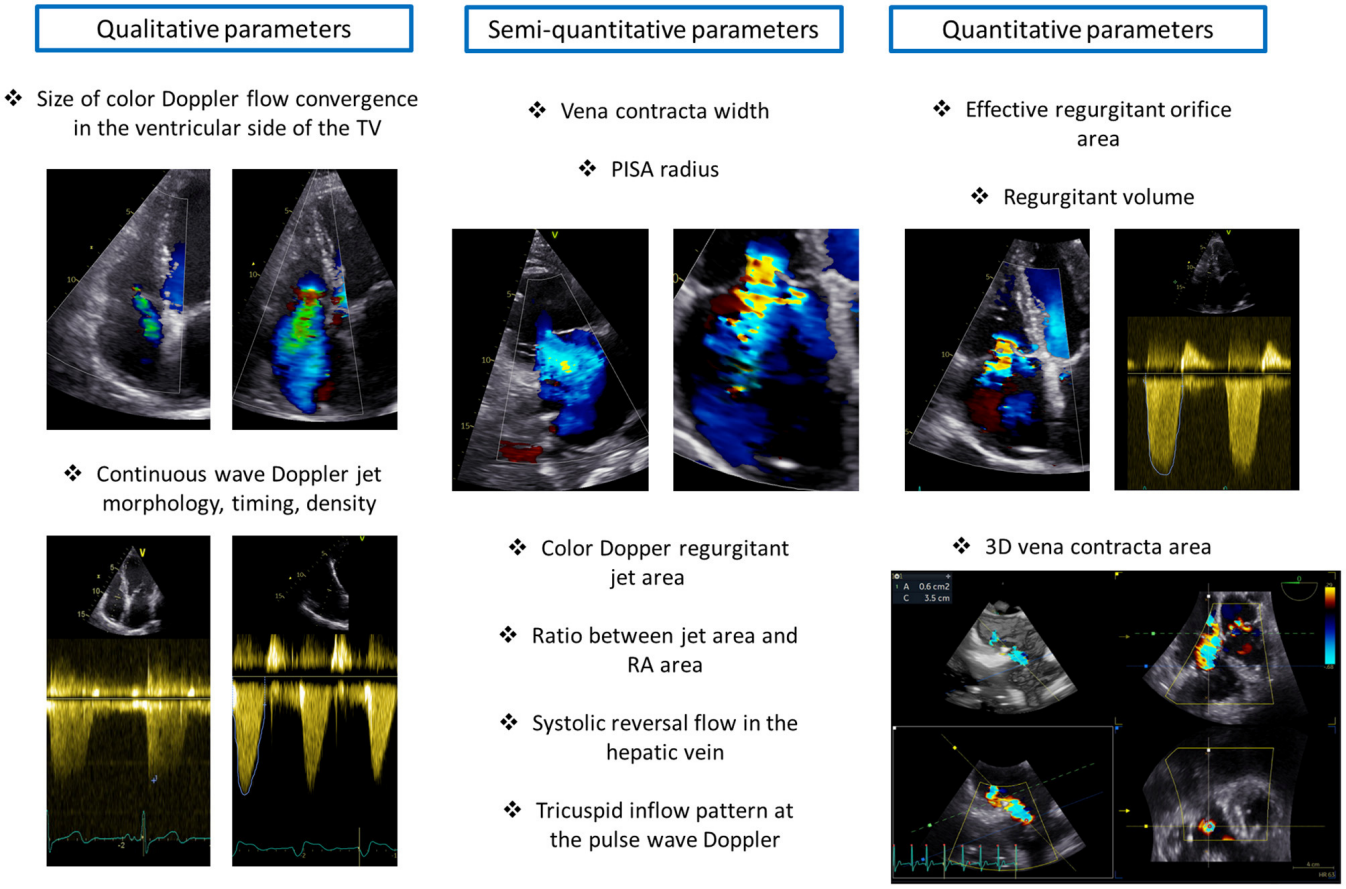
Grading of tricuspid regurgitation severity. The figure illustrates the main qualitative, semi-quantitative and quantitative parameters of tricuspid regurgitation severity. PISA, proximal isovelocity surface area; RA, right atrium; TV, tricuspid valve.

Echocardiography represents the mainstay imaging modality to grade TR severity (26). According to current recommendations (26), this evaluation should be based on multiple parameters related not only to the TR but also to the RA and RV. TR can be determined by RA and/or RV dilatation but can also be the cause of RV and RA dilatation through chronic volume overload and increased wall tension (35). Therefore, both RA and RV size should be taken into account when grading TR. The echocardiographic parameters of TR severity can be grouped into three categories: qualitative, semi-quantitative, and quantitative (26) (Figure 7).

The qualitative parameters of TR severity are not based on mathematical formulas but mainly on the subjective assessment of the physician and therefore are largely dependent on experience and come with limitations. A large color Doppler flow convergence in the ventricular side of the TV throughout the systole suggests severe TR. However, this parameter is highly dependent on the settings of the machine, loading conditions and can underestimate TR severity in the presence of eccentric jets. Moreover, the density, timing and shape of the continuous wave Doppler jet of TR can be informative on TR severity. A dense, holosystolic, with an early peak TR jet is suggestive of severe TR.

Semi-quantitative parameters of TR severity provide a more detailed picture on TR severity compared to a qualitative assessment. A color Doppler TR jet area > 10 cm^2^ indicates severe TR and the same applies to a ratio between the regurgitant jet area and RA area > 33%. The vena contracta (VC) width is another semi-quantitative parameter of TR severity and represents the diameter of the narrowest portion of the regurgitant jet immediately downstream of the regurgitant orifice. A VC width ≥ 7 mm is consistent with severe TR. A proximal isovelocity surface area (PISA) radius ≥ 9 mm (measured form the apical four-chamber view with an aliasing velocity of ~28 cm/s) is also indicative of severe TR. The presence of systolic reversal flow in the hepatic vein (sampled with pulse wave Doppler) represents a specific but not sensitive parameter of severe TR because it is largely affected by the direction of the jet and by the presence of atrial fibrillation. Finally, the evaluation of the tricuspid inflow pattern with the pulsed-wave Doppler can indicate the severity of TR. A prominent E wave (>1.0 m/s) is usually present in patients with severe TR; nevertheless, the tricuspid inflow pattern is highly affected by the cardiac rhythm and should not be taken into account as an index of TR severity in patients with atrial fibrillation.

The quantitative parameters of TR severity give a quantitative estimation of TR severity based on several geometrical and mathematical assumptions that may not be met in some cases. These parameters include the estimation of the effective regurgitant orifice area (EROA) and regurgitant volume based on the PISA method. Although, EROA can be underestimated in the presence of an elliptic orifice, recent studies have shown an important relation of this parameter with prognosis in patients with significant TR (36). An EROA ≥ 40 mm^2^ and a regurgitant volume ≥ 45 ml/beat are suggested by the guidelines to identify severe TR (26).

Three-dimensional echocardiography with multiplanar reconstruction can also be informative on the severity of TR. However, despite the potential advantages of 3D echocardiographic parameters of TR severity, such as VC area (37), further studies are needed to inform on the added value of these measurements and to provide cut-off values to be used in clinical practice. Moreover, recent studies showed that patients referred for TV transcatheter interventions often present with a TR that is far above the lower threshold currently recommended to identify severe TR (38, 39) highlighting the need for a novel grading system that could depict the whole spectrum of TR severity (40). A group of experts proposed the introduction of two new TR categories, massive and torrential TR, by extending the current cut-off values for severe TR (41, 42). The value of these new TR categories defined by the use of individual parameters of TR severity and of a quantitative assessment had been shown in several studies (36, 38, 42, 43), and recently also the systematic combined use of VC width and EROA to identify severe (VC width ≥ 7 mm and EROA <80 mm^2^) and torrential TR (VC width ≥ 7 mm and EROA ≥ 80 mm^2^) showed to be useful to predict patient outcomes in patients with significant secondary TR (44). When reporting an echocardiographic exam of a patient with significant TR, quantitative and semi-quantitative parameters of TR severity should always be recorded to allow for comparisons in follow-up exams and to objectively evaluate the effect of medical and interventional therapies on TR severity.

MDCT can provide some indirect suggestions on TR severity. Although direct evaluations of the regurgitant orifice are rarely feasible, some qualitative and quantitative indirect parameters can be indicative of the severity of TR and be useful if the echocardiographic judgment is not consistent between different parameters or observers. The level of opacification of the RA during systole or the early opacification of the inferior vena cava or hepatic veins have been described as specific indirect indicators of TR severity (45). The calculation of the regurgitant volume as the difference between LV and RV stroke volume can be informative on TR severity in the absence of significant concomitant valvular heart diseases. The TA area measured in mid-systole can provide important suggestions on the etiology and severity of TR (46).

CMR has been considered the reference imaging technique for quantitative valvular assessment and has recently gained a lot of attention for the characterization of TR severity. This imaging modality allows for the accurate quantification of the regurgitant volume by subtracting the pulmonic forward volume from the RV stroke volume. It is also possible to calculate the regurgitant fraction as the ratio between the TR regurgitant volume and RV stroke volume. Currently the same cut-off values to classify mitral regurgitation are recommended for the abovementioned parameters (47). Nevertheless, considered the frequent disagreement between echocardiography and CMR in classifying the severity of TR (48), a validation against patient outcomes would be needed. Finally, the direct visualization of the regurgitant orifice has also been described (49); however, the direct visualization of the TV may be very challenging with CMR and further data would be needed before considering the implementation of this evaluation in clinical practice.

## Assessment of Right Ventricular Remodeling and Systolic Function

TR causes a chronic volume overload on the RV and RA which leads to further dilatation and dysfunction of the right heart chambers. RV remodeling, consisting of RV dilatation and/or dysfunction, should always be assessed in patients with significant TR as it may be the cause of TR, may be indicative of the severity of TR, represents an important parameter to guide decision-making regarding interventions (9, 10), and can significantly affect patient prognosis (50). The RV has a complex, irregular structure with a conical shape in the longitudinal plane and a crescent shape in the sagittal plane. Moreover, the TV and pulmonary valve are not in continuity and is difficult to image them on the same plane. Three portions of the RV can be identified: the inlet (that starts with the TV), the apical trabecular portion and the outlet (that ends with the pulmonary valve) (Figure 1). Therefore, multiple views are necessary to characterize RV dimensions when using 2D echocardiography (27) (Figure 4). The long-axis, short-axis and RV inflow views should be acquired from the parasternal window. From the apical window, the four-chamber and the RV focused views should also be acquired. Finally, from the subcostal window a four-chamber and short-axis view can be acquired. According to current recommendations, the RV dimensions should be assessed from an apical four-chamber view, focused on the RV, at end-diastole and care should be taken to acquire the image that demonstrates the maximum diameters without foreshortening the RV (27). This can be confirmed by the presence of the crux and apex of the heart simultaneously in view. An RV basal diameter ≥ 42 mm, a mid-ventricular diameter ≥ 35 mm, and a base-to-apex length ≥ 86 mm are all indicative of RV dilatation (27). The pattern of RV, TA, and RA remodeling is suggestive of the cause of TR. A more pronounced dilatation at the level of the mid-RV diameter is suggestive of pulmonary hypertension and may be associated with marked TV leaflet tethering due to the apical displacement of the RV papillary muscles (51). On the contrary a more pronounced dilation of the TA and RA in a patient with atrial fibrillation may indicate an important role of the RA in causing TR through TA enlargement and TV leaflet malcoaptation (52). Moreover, the RV should be compared to the left ventricle to gain a better understanding of the pathophysiological mechanism of TR. An LV end-systolic or end-diastolic eccentricity index, evaluated from the short-axis view, > 1.1 (that indicates a compression of the RV on the left ventricle) has been described as suggestive of pulmonary hypertension (53). However, a more pronounced compression of the RV on the LV at late diastole (as compared to the one in systole) has been described as suggestive of predominant RV chronic volume overload due to TR (54).

**Figure 8:**
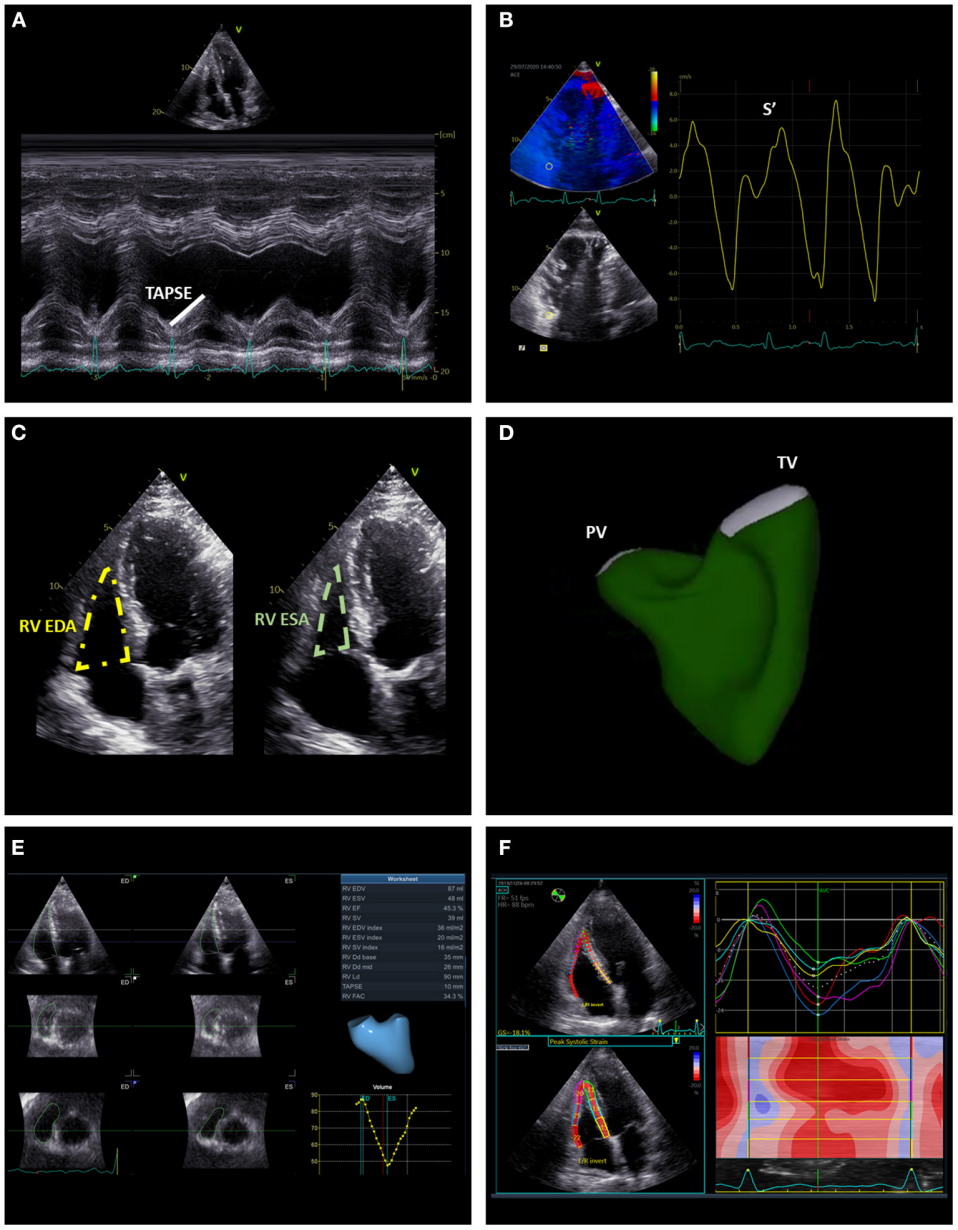
Summary of the main echocardiographic parameters of right ventricular systolic function. The figure illustrates conventional [TAPSE **(A)**, S' **(B)**, and FAC **(C)**] and advanced [RV EF **(D,E)**, global and free-wall RV longitudinal strain **(F)**] echocardiographic indices of RV systolic function. EDA, end-diastolic area; EDV, end-diastolic volume; ESA, end-systolic area; ESV, end-systolic volume; RV, right ventricle; TAPSE, tricuspid annular plane systolic excursion.

The tricuspid annular plane systolic excursion (TAPSE), the systolic excursion velocity of the lateral portion of the TA (S') and RV fractional area change (FAC) are the most widely used standard 2D echocardiographic parameters of RV systolic function (Figure 8). TAPSE is obtained by aligning the M-mode cursor to the lateral aspect of the TA from an apical four-chamber view and measuring the amount of the longitudinal motion of the annulus at peak systole (27). A value of TAPSE < 17 mm is indicative of RV systolic dysfunction. This parameter is very simple and showed to be independently related with prognosis in patients with secondary TR (50). Nevertheless, the use of TAPSE as a marker of RV systolic function is based on the assumption that one segment can represent the function of a complex 3D structure (i.e., the RV) and this may not be the case in certain conditions (for instance, after cardiac surgery) (55). RV S' is acquired by placing the sample volume of the pulsed wave Doppler either at the lateral portion of the TA or the basal segment of the RV and is read as the highest systolic peak. An S' < 10 cm/s indicates RV systolic dysfunction. Similar to TAPSE, this is a simple parameter of RV systolic function which comes from the assessment of a single segment of the RV that may be not representative of the whole RV chamber. RV FAC is the percent change of the RV area between end-diastole and end-systole evaluated from an apical four-chamber view. An FAC < 35% is suggestive of systolic dysfunction. Although, FAC correlated well with RV ejection fraction evaluated with CMR (56), it is dependent on image quality and the 2D areas of the RV may not be representative of RV dimensions especially if complex RV remodeling had occurred.

All the conventional echocardiographic parameters are highly affected by loading conditions and may not be representative of RV contractility. Speckle-tracking echocardiography (STE) is angle independent and the STE-derived global and free-wall longitudinal strain are less load dependent parameters of RV systolic function (Figure 8). RV global and free-wall strain represent the percentage change in myocardial deformation of these areas during systole. RV free-wall strain showed an independent association with all-cause mortality in patients with significant secondary TR and incremental prognostic value over FAC and TAPSE (57). Recently, STE-derived RV global longitudinal strain data have been combined with echocardiography-derived RV pressures to obtain RV myocardial work indices and these indices have been compared with invasive hemodynamics of the RV in patients with heart failure with reduced ejection fraction (58). In this study, none of the conventional echocardiographic indices of RV systolic function correlated with stroke volume and stroke volume index. On the contrary, RV global constructive work (which is an index of the RV work functional to contraction during systole and relaxation during diastole) showed a moderate correlation with stroke volume and stroke volume index potentially indicating the importance of taking the RV afterload into account when analyzing RV systolic function (58). However, the value of these indices in patients with significant TR has not yet been investigated.

The complex RV geometry and systolic function can be better assessed with 3D imaging techniques. Although still challenging by suboptimal quality of the acoustic window, 3D echocardiography can be useful for an accurate assessment of RV volumes and to calculate RV ejection fraction (Figure 8). An excellent correlation has been demonstrated between 3D echocardiography and CMR for the assessment of RV volumes and ejection fraction (59). Of note, 3D echocardiography tends to underestimate RV volumes compared to CMR, and also a slight underestimation of RV ejection fraction had been suggested (60). CMR is considered the gold standard for the assessment of RV size and systolic function (Figure 6). Nevertheless, similarly to what had been described for the LV in the presence of mitral regurgitation (61), RV ejection fraction may largely overestimate RV systolic function in the presence of significant TR. From this perspective, theoretically, feature-tracking derived RV global or free-wall longitudinal strain using CMR (62) may provide a better estimation of RV contractility compared to ejection fraction when significant TR is present. However, the use of CMR feature-tracking parameters has yet to be investigated in this specific cohort. The RV mass and also the stress induced by the RV on the interventricular septum (expressed by the T1 mapping measured in the short axis at upper and lower RV insertion points) are valuable indices of RV remodeling especially in patients with pulmonary hypertensions and can be evaluated with CMR (63, 64) (Figure 6). Finally, full-beat MDCT can also be used to characterize RV volumes, ejection fraction and myocardial deformation (65) (Figure 5) but considered the need of radiation exposure, CMR is usually preferred for the evaluation of RV remodeling.

## Specific Procedures on the Tricuspid Valve and Additional Imaging Data Needed for Pre-Procedural Planning

Different surgical and percutaneous options are currently available for the treatment of TR and, similarly to other valvular heart disease (66), multimodality imaging is key to select the best option and timing for the patient and provide all the data needed to adequately plan the procedure and prevent complications.

In patients undergoing cardiac surgery for other indications or not at high surgical risk, surgery represents a valuable option to treat TR. Overall, surgical TV repair should be preferred over TV replacement because, when feasible, it is associated with less complications and better outcomes (67). The aim of TV repair should be to reduce the TA diameter and restore leaflet coaptation. The best repair technique for the patient should be chosen based on an accurate pre-procedural assessment that relies mainly on echocardiography. The exact mechanism of TR should be identified in order to properly correct it during surgery. The TA diameter can be reduced with a ring annuloplasty. The ring has the function of reducing the TA diameter and stabilizing it in systole. The size of the ring can be chosen based on the TA diameters measured with 2D-3D echocardiography (Figure 4) and confirmed with intra-procedural commercial tricuspid sizers (68). In the presence of significant TV leaflet tethering detected and quantified with echocardiography, a ring annuloplasty may not be sufficient, and other techniques should be considered such as the augmentation of the anterior leaflet with a pericardial patch or stitching the free edges of the TV leaflets with the clover technique (69). If the TR is primary and due to a prolapsing leaflet with redundant tissue, a triangular resection can be considered, while, if the prolapse is due to chordal rupture, a chordal transposition or the implantation of artificial chordae can be performed (69). Finally, if the excessive systolic motion of one or more TV leaflets is due to chordal elongation, a sliding plasty lowering the location of the corresponding papillary muscle can be performed (69). Transesophageal echocardiography plays a major role in the operating theater also to assess the residual TR and the need of further corrections.

In patients at high or prohibitive surgical risk or without the need of other cardiac surgeries, a transcatheter procedure to treat TR can be considered. Different options exist and again the choice should be based on the pre-procedural assessment and identification of the mechanism of TR, mainly using echocardiography. In patients where the dilation of the TA is the main mechanism causing TR, a transcatheter direct annuloplasty represents a valuable option and three systems are currently available: the Trialign system, the TriCinch device, and the Cardioband system. The Trialign is a suture based TV direct annuloplasty system that reduces the TA diameter through tissue plication (70). From a trans-jugular access, two pledgeted sutures are placed at the level of the antero-posterior and postero-septal commissures on the TA then the pledgets are approximated to each other causing the shrinkage of the posterior leaflet and the bicuspidization of the TV. A 2D/3D transesophageal echocardiographic exam is crucial to assess that enough space is present on the posterior portion of the TA to place the two pledgeted sutures (70). The percutaneous TV annuloplasty system for symptomatic chronic functional tricuspid regurgitation (SCOUT) trial confirmed the feasibility and efficacy of the Trialign device in reducing the TA dimensions, TR severity and in improving stroke volume and quality of life 30-day after the procedure (39). The TriCinch system comprises of two components: (i) a stainless steel corkscrew implant that has to be placed on the TA at the level of the antero-posterior commissure; and (ii) a self-expanding nitinol stent that is implanted on the inferior vena cava to stabilize the system (71). This system is tensioned to reduce the TA dimensions and TR. During the 1st year of follow-up in the percutaneous treatment of TV regurgitation with the TriCinch system (PREVENT) trial, five patients (23%) experienced late anchor detachments (72). To overcome this issue, the design of the device had been improved and is now under evaluation.

**Figure 9:**
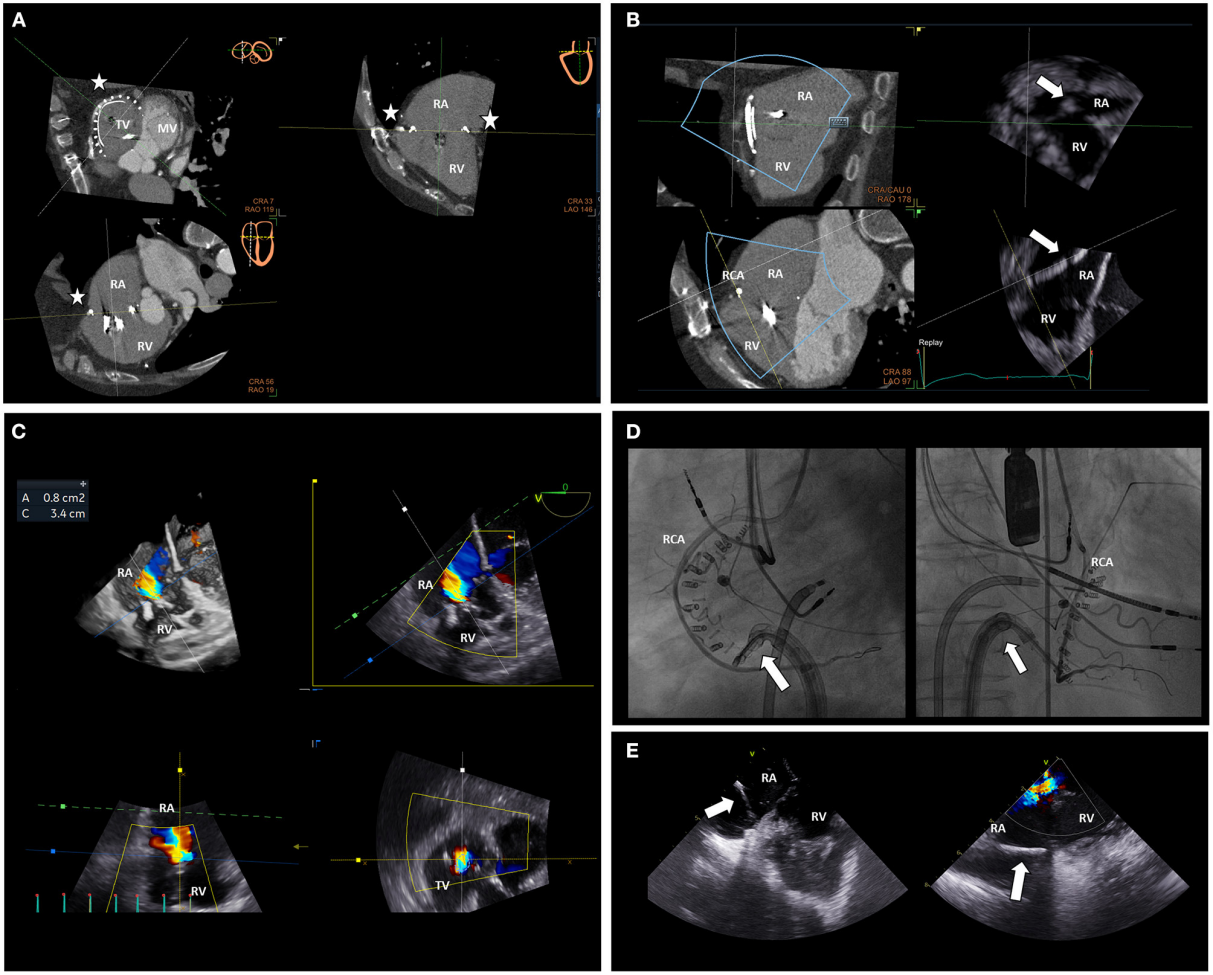
Multimodality imaging for intra-procedural guidance of a Cardioband procedure on the tricuspid valve. MDCT data displaying the predicted position of the anchors of the Cardioband device (stars) can be aligned with real-time echocardiographic data **(A)** during the procedure. The MDCT-echocardiography fusion imaging permits a better alignment of the delivery system (arrow) to place the anchors in the predicted position on the TA **(B)** avoiding potential complications such as RCA compression or damage. TR severity and TA dimensions can be assessed during the procedure with multi-planar reconstruction echocardiography **(C)** to monitor the result. Orthogonal fluoroscopic views can be used to locate the delivery system (arrows), the anchors of the Cardioband device and their relative position to the RCA **(D)**. Moreover, intracardiac echocardiography can be very helpful to align the delivery system (arrows) to the TA and aim for an optimal position of the anchors **(E)**, especially in the case of challenging transesophageal echocardiographic views or difficult anatomy. MV, mitral valve; RA, right atrium; RCA, right coronary artery; RV, right ventricle; TV, tricuspid valve.

The Cardioband system is the percutaneous equivalent of the surgical undersized annuloplasty. This system consists of an adjustable, sutureless annuloplasty band of polyester through which anchors are inserted (13). The system is delivered through a transfemoral approach and the anchors are implanted on the atrial side of the TA from the antero-septal through the postero-septal commissure. After placing the anchors, the size of the system can be reduced to reduce the TA dimensions, under real-time transesophageal echocardiography monitoring, and improve TV leaflets coaptation until obtaining a significant reduction of TR (Figures 5, 9). An accurate pre-procedural planning is necessary and requires the use of echocardiography and MDCT. Transthoracic and transesophageal 2D and 3D echocardiography can provide information on the mechanism of TR, TA dimensions, TV leaflet integrity, RV/RA remodeling and identify the best transesophageal views that would be necessary for the real-time monitoring during the procedure. MDCT is used to assess the feasibility of the procedure (Figure 5). A severely calcified TA can interfere with the implantation of the anchors of the device and is considered a contraindication. The distance between the RCA and the TA can be assessed with contrast-enhanced MDCT using specific protocols to verify the presence of enough tissue on the TA to implant the anchors, identify the best location for the anchors and prevent damage or compression of the RCA during the procedure (18) (Figure 5). The high spatial resolution of MDCT allows also the reliable assessment of TA dimensions to choose the right device size. Moreover, a post-processing software permits to use MDCT data to reproduce transesophageal echocardiographic views that can be useful to plan the imaging of the procedure (32). The 6-month follow-up results of the tricuspid regurgitation repair with Cardioband transcatheter system (TRI-REPAIR) trial showed the safety and efficacy in reducing the TA dimensions, TR severity and improving heart failure symptoms and quality of life of the Cardioband system in patients with moderate or severe TR (73).

Another percutaneous option to repair the TV is represented by the devices that can restore the TV leaflet coaptation. The Triclip system is an adaptation of the Mitraclip system to the TV. Both the Mitraclip and the Triclip systems can be used to achieve a percutaneous edge-to-edge repair of the TV and are useful to treat either primary or secondary TR (74). Two sizes of clip can be implanted, the 4 mm (size NTR) or the 7 mm (size XTR) which can be useful for larger coaptation gaps. A comprehensive transthoracic and transesophageal 2D and 3D echocardiographic exam is required to assess the feasibility of the procedure and identify the number, type and ideal location for the clips. The TV leaflets length, integrity, tethering and coptatation gaps should be evaluated and recorded. The TV leaflets should be ≥ 6 or ≥ 9 mm to allow the grasping of the NTR and XTR, respectively (75). Moreover, the coaptation gaps should be ≤ 7 mm (75). A larger coaptation gap, tenting area and EROA are all identified predictors of procedural failure (76). The PASCAL system is another alternative to perform a tricuspid edge-to-edge percutaneous repair (77). This system is constituted by a clip and a spacer making it potentially useful for larger TA and coaptation gaps. The FORMA spacer system is another device that can improve the leaflet coaptation with the implantation of a spacer at the level of the coaptation gap of the TV. It is constituted by two components: (i) a foam filled balloon (the spacer) which is positioned at the level of the coaptation gap and offers a coaptation surface to the TV leaflets, and a (ii) rail which is anchored to the RV apex to stabilize the device. It is available in different sizes that are chosen based on the largest VC width measured with echocardiography (78). Nevertheless, further studies are needed to confirm its safety and efficacy and is not yet available for medical use.

Transcatheter TV replacement represents a valuable option in the case of failing TV annuloplasty or biological TV prosthesis. Prostheses normally applied in the aortic position have been successfully used for valve-in-ring or valve-in-valve procedures on failing TV annuloplasty or biological TV prosthesis (79). A meticulous pre-procedural planning using echocardiography and MDCT is determinant to achieve a successful implant. The degeneration and stability of the previous surgical TV replacement or repair should be assessed with echocardiography as well as the presence of paravalvular leaks that can make the transcatheter TV replacement not indicated. MDCT can be used to identify the size of the ring or prosthesis previously implanted and choose the right size and landing zone for the new prosthesis.

Finally, transcatheter valves can be implanted in the superior and/or inferior caval veins to reduce the backflow of blood from the incompetent TV to the caval veins and reduce symptoms and signs of venous congestion. The implantation of balloon-expandable valves requires pre-stenting of the inferior vena cava to downsize the landing zone and ensure safe anchoring of the transcatheter valve (80). On the contrary, self-expandable valves can be specifically designed to fit the size of inferior or superior vena cava and do not require pre-stenting of the landing zone (81). Echocardiography can help to identify patients with severe TR and systolic backflow on the hepatic veins who are more likely to benefit from these procedures. Of note, the implantation of transcatheter valves in the inferior and/or superior vena cava does not reduce the chronic volume overload on the RV; therefore, a careful evaluation of RV function (Figure 8) should be performed before the procedure, as patients with RV systolic dysfunction are less likely to benefit from it. Accurate measurement of the dimensions of the caval veins should be performed for the sizing of the transcatheter valves to avoid the risk of valve migration and choose the best location for the landing zone (11).

## Intraprocedural Guidance of Percutaneous Procedures on the Tricuspid Valve

Similar to transcatheter procedures on heart valves other than the TV, intraprocedural guidance of percutaneous procedures on the TV relies on multimodality imaging (Figure 9). 3D transesophageal echocardiography plays a key role in the real-time monitoring of these procedures. Nevertheless, compared to the mitral valve, the TV is located more anteriorly and far from the transesophageal echocardiographic probe which can make it challenging to visualize certain locations on the TA especially during direct transcatheter annuloplasty. The presence of pace-maker or implantable cardioverter defibrillator lead can create acoustic shadows that can cover some important structures. 3D echocardiography and simultaneous biplane visualization may improve spatial orientation when maneuvering the catheters on the right heart or when placing the anchors of the Cardioband device. Differently from echocardiography, fluoroscopy does not provide any information on the soft tissue but can be very important to guide the catheters through the TV and usually two orthogonal views are used (Figure 9). The two fluoroscopic views are generally patient-specific and can be predicted from the MDCT data (13). The first one is usually the cranial right anterior oblique view displaying the long-axis view of the RV and is used to verify the trajectory of the delivery system of the anchors compared to the orientation of the TA and to verify the advancement of the anchors while they are placed. The second fluoroscopic view is usually the caudal left oblique view where the TV can be observed from the RV and can be compared to the “en face” 3D view of the TV obtained with transesophageal echocardiography; this view is used to improve the orientation of the delivery system. To prevent complications, a guide can be placed to identify the trajectory of the RCA in the fluoroscopic views. The fluoroscopic and echocardiographic data can be fused into the same image with a dedicated software to increase the integration of these data and improve the communication between imagers and interventionalists during the procedure (82). More recently, pre-procedural MDCT data were also be combined with real-time echocardiographic data by a dedicated software to ascertain that the position where the anchors are placed corresponds with the pre-planned ones (83) and avoid potential complications (Figure 9). Intracardiac echocardiography may help for the visualization of the TA during transcatheter TV direct annulopasty (especially for the placement of the most posterior anchors) and to ensure adequate orientation and grasping of the leaflets during transcatheter TV edge-to-edge repair (84), particularly in patients with a difficult anatomy or acoustic window (Figure 9). Finally, transesophageal echocardiography allows for real-time assessment of the final result, residual TR and complications that may require urgent/emergent treatment such as pericardial effusion or cardiac tamponade.

## Follow-Up Imaging After Interventional Procedures on the Tricuspid Valve

Transthoracic 2D/3D echocardiography is the main imaging modality used for the follow-up of patients after surgical or percutaneous procedures on the TV. Several information should be assessed and recorded. First, it is very important to assess residual TR before discharging the patient as it may differ from the evaluation performed in the immediate post-procedural phase in the operating theater where the patient might have been under general anesthesia, invasive ventilation or sedation with different RV loading conditions. Qualitative, semi-quantitative and, most importantly, quantitative parameters of TR severity should be recorded (Figure 7) to allow for objective comparisons during the follow-up. RV remodeling (Figure 4), systolic function (Figure 8) together with TA dimensions should also be reported. It is important to remember that after cardiac surgery with pericardial opening the longitudinal function of the RV usually worsens (85) but the radial RV contraction should recover. The presence of potential short and late-term complications such as device detachment, degeneration, annular or leaflet tear, endocarditis, pericardial effusion or cardiac tamponade should be detected with transthoracic echocardiography, and in case of dubious findings a transesophageal 2D/3D echocardiographic exam or, as a second step, an MDCT may be performed to elaborate on and clarify the findings.

## Conclusions

The accumulating evidence on the negative prognostic impact of TR and the development of effective transcatheter procedures to treat also patients with multiple comorbidities and at high or prohibitive surgical risk have increased the interest on the imaging of the right heart and TV. Multimodality imaging plays a key role to assess TR severity, TA dimensions, RV and RA remodeling, define the etiology of TR, choose the timing and the best therapeutic option, guide the procedure, prevent complications and follow-up patients with significant TR after interventions.

## Author Contributions

FF wrote the first draft of the manuscript and prepared the figures and adjusted it according to the comments and suggestions of the co-authors. KH helped in preparing the figures and critically revised the manuscript. JB and VD critically revised the manuscript. NA helped in structuring the manuscript, preparing the figures, and critically revised the manuscript. All authors contributed to the article and approved the submitted version.

## Conflict of Interest

The Department of Cardiology of the Leiden University Medical Center received research grants from Abbott Vascular, Bayer, Bioventrix, Medtronic, Biotronik, Boston Scientific, GE Healthcare, Ionis, and Edwards Lifesciences. JB received speaking fees from Abbott Vascular. VD received speaker fees from Abbott Vascular, Medtronic, Edwards Lifesciences, Novartis, MSD, and GE Healthcare. NA received speaker fees from Abbott Vascular and GE Healthcare. The remaining authors declare that the research was conducted in the absence of any commercial or financial relationships that could be construed as a potential conflict of interest.
